# Extreme ambient temperatures and cardiorespiratory emergency room visits: assessing risk by comorbid health conditions in a time series study

**DOI:** 10.1186/1476-069X-13-5

**Published:** 2014-02-03

**Authors:** Eric Lavigne, Antonio Gasparrini, Xiang Wang, Hong Chen, Abderrahmane Yagouti, Manon D Fleury, Sabit Cakmak

**Affiliations:** 1 Environmental Issues Division, Public Health Agency of Canada, Ottawa, Canada; 2 Department of Medical Statistics, London School of Hygiene and Tropical Medicine, London, UK; 3 Public Health Ontario, Toronto, Canada; 4 Dalla Lana School of Public Health, University of Toronto, Toronto, Canada; 5 Institute for Clinical Evaluative Sciences, Toronto, Canada; 6 Climate Change and Health Office, Health Canada, Ottawa, Canada; 7 Population Studies Division, Health Canada, Ottawa, Canada

**Keywords:** Temperature, Climate change, Cardiovascular, Respiratory, Comorbidity

## Abstract

**Background:**

Extreme ambient temperatures are an increasing public health concern. The aim of this study was to assess if persons with comorbid health conditions were at increased risk of adverse cardiorespiratory morbidity during temperature extremes.

**Methods:**

A time series study design was applied to 292,666 and 562,738 emergency room (ER) visits for cardiovascular and respiratory diseases, respectively, that occurred in Toronto area hospitals between April 1st 2002 and March 31st 2010. Subgroups of persons with comorbid health conditions were identified. Relative risks (RRs) and their corresponding 95% confidence intervals (CIs) were estimated using a Poisson regression model with distributed lag non-linear model, and were adjusted for the confounding influence of seasonality, relative humidity, day-of-the-week, outdoor air pollutants and daily influenza ER visits. Effect modification by comorbid health conditions was tested using the relative effect modification (REM) index.

**Results:**

Stronger associations of cardiovascular disease ER visits were observed for persons with diabetes compared to persons without diabetes (REM = 1.12; 95% CI: 1.01 – 1.27) with exposure to the cumulative short term effect of extreme hot temperatures (i.e. 99^th^ percentile of temperature distribution vs. 75^th^ percentile). Effect modification was also found for comorbid respiratory disease (REM = 1.17; 95% CI: 1.02 – 1.44) and cancer (REM = 1.20; 95% CI: 1.02 – 1.49) on respiratory disease ER visits during short term hot temperature episodes. The effect of extreme cold temperatures (i.e. 1^st^ percentile of temperature distribution vs. 25th percentile) on cardiovascular disease ER visits were stronger for individuals with comorbid cardiac diseases (REM = 1.47; 95% CI: 1.06 – 2.23) and kidney diseases (REM = 2.43; 95% CI: 1.59 – 8.83) compared to those without these conditions when cumulated over a two-week period.

**Conclusions:**

The identification of those most susceptible to temperature extremes is important for public health officials to implement adaptation measures to manage the impact of extreme temperatures on population health.

## Background

Climate change will result in an increase in temperature extremes [1], which raises important concerns for public health [2]. Several epidemiological studies have reported that extreme hot and cold temperatures are associated with an increase in daily mortality and morbidity [3-5]. Growing evidence from recent studies suggests that persons with pre-existing medical conditions such as diabetes, cardiovascular diseases and respiratory diseases may be at increased risk of mortality during extreme temperature episodes [6,7]. However, less is known about medical conditions that confer susceptibility to cardiorespiratory morbidity during extreme temperature episodes.

Previous studies in Canada have shown health impacts in the days following exposure to cold and hot temperatures [8-11]. Although no previous study investigated comorbidity factors that increase susceptibility to temperature extremes in Canada, previous studies in other parts of the world have reported that pre-existing medical conditions such as diabetes, respiratory diseases and cardiovascular diseases may increase the risk of death during hot and cold days [6,7,12,13]. However, most research to date has investigated susceptibility to temperature extremes on mortality, with less attention in identifying vulnerability factors to temperature on morbidity outcomes.

The aim of this study was to evaluate if persons with comorbid health conditions were at increased risk of adverse cardiorespiratory morbidity during extreme hot and cold temperatures compared to individuals without comorbid health conditions. A time series study was applied using daily emergency room visits for the treatment of respiratory and cardiovascular diseases in Toronto area emergency departments. Subgroups of persons with comorbid health conditions were identified. Toronto is the largest city in Canada with 2.7 million residents [14] and it is located in a temperate climate zone with a wide range of hot and cold temperatures throughout the year.

## Materials and method

### Study population and health outcome data

The study population included residents of Toronto who were admitted to an emergency department within the city from April 1^st^ 2002 to March 31^st^ 2010 with main diagnoses of cardiovascular (International Classification of Diseases, 10^th^ Revision [ICD-10] codes: I00-I99) or respiratory diseases (ICD-10 codes: J00-J99). The daily numbers of ER visits were identified through the National Ambulatory Care Reporting System (NACRS) [15] using the first three digits of available postal codes corresponding to the city of Toronto [15]. The NACRS database is estimated to capture more than 97% of the emergency department visits in Toronto and has been demonstrated to capture accurately the coding of the most responsible diagnosis when being admitted to the ER [16]. We also tabulated the daily number of visits for influenza (ICD-10: J09, J10, J11) in order to adjust for the potential confounding influence of viral respiratory seasonal epidemics in the analyses using respiratory disease ER visits as an outcome variable. Access to the NACRS database was granted through data sharing agreement between the Canadian Institute for Health Information (CIHI) and the Public Health Agency of Canada (PHAC).

### Comorbid health conditions

The comorbid health conditions for each ER visit were defined by using all secondary ICD-10 diagnosis codes listed for the same visit as the main diagnosis of cardiovascular or respiratory diseases. The comorbid health conditions that were extracted from the secondary diagnoses are defined as follows: diabetes (ICD-10 codes: E10-E14), chronic respiratory diseases (ICD-10 codes: J40-J47), upper respiratory infections (ICD-10 codes: J00-J06), hypertension (ICD-10 code: I10), cancer (ICD-10 codes: C00-D49), kidney diseases (ICD-10 codes: N17-N19), pneumonia (ICD-10 codes: J12-J18) and cardiac diseases (ICD-10 codes: I01, I02.0, I05–I09, I11, I13, I20–I25, I27 and I30–I52). Secondary diagnoses of chronic respiratory diseases and cardiac diseases were validated by linking the data with the Hospital Morbidity Database (HMD) which captures all hospitalization in Canada and with the NACRS database. One physician service one year prior to the ER visit in either the HMD or NACRS for the medical condition under consideration was considered for identifying the comorbid conditions [8]. A secondary diagnosis of cancer was validated by linking the data with the Canadian Cancer Registry which captures all diagnoses of cancer across Canada. A one year time window before the date of ER visit was used to identify subjects with cancer. Each case of cardiovascular or respiratory disease ER visits was categorized in subgroups in a time series dataset.

### Weather and air pollution data

Daily weather data were obtained from Environment Canada using the monitoring station at Toronto Pearson International Airport (latitude: 43°40′36″N; longitude: 79°37′50″W) located approximately 26 km west of downtown Toronto. Daily averages of temperature and relative humidity were computed based on hourly measurements.

Daily ambient concentrations of air pollution were obtained from the National Air Pollution Surveillance Network (NAPS) maintained by Environment Canada which collects outdoor air pollution levels through several automated fixed-site monitoring stations [17]. Daily average concentrations for the following pollutants were obtained: nitrogen dioxide (NO_2_), sulphur dioxide (SO_2_), carbon monoxide (CO), ozone (O_3_) and particulate matter of median aerodynamic diameter less than 2.5 microns (PM_2.5_). Daily values were computed by averaging across all monitoring stations the daily mean concentrations of each pollutant for each day. A minimal amount of missing values was observed for O3 and CO. We interpolated the missing values for these pollutants by using the mean of previous and the following days’ concentration.

### Statistical analysis

The association between extreme ambient temperatures and daily numbers of cardiovascular ER visits or respiratory ER visits according to subgroups of comorbid health conditions was assessed using a time series study design. A quasi-likelihood Poisson regression in a generalized linear model (GLM) [18] was fitted to model the effect of daily mean temperature on the daily numbers of cardiovascular and respiratory ER visits by potentially sensitive subgroups. Daily mean temperature was chosen as the main exposure metric because it represents the exposure throughout the whole day and can be easily used for decision making purposes [19,20]. In addition, a distributed lag non-linear model (DLNM) [21,22] was incorporated in the Poisson regression in order to explore the non-linear relationships between temperature and daily ER visit counts cumulated across specific lag periods. We investigated effect modification by comorbid health conditions by calculating the relative effect modification (REM) index which is the ratio between the relative risk (RR) when comorbidity is present and when the comorbid health condition is absent (reference category) [23]. The REM can be interpreted as the relative increase in risk for an emergency room visit for persons with a comorbid health condition compared to those without the health condition. The statistical significance of the REM was tested with the calculation of its 95% confidence interval.

We adjusted for seasonal effects and long-term trends using a natural cubic spline for time with 10 degrees of freedom (*df*) per year and we accounted for sub-seasonal cycles by including an indicator variable for day-of-the-week. Several other smoothers for time were also assessed (e.g. 5, 7, 9, 11 and 13 *df*), but the optimal fit was suggested to be 10 *df* per year. A natural cubic spline was used in the DLNM with 5 *df* for the non-linear temperature effects and 5 *df* for the lagged effect. Spline knots were placed at equal spaces across the range of daily mean temperature as well as at logarithmically equal intervals in the lag space. A 21 day lag period was used to examine the effect of temperature on ER visits [24]. We also investigated adjustment for 0 to 2 lag days of moving average (i.e. average of the concurrent day and the two previous days) of specific air pollutants (NO_2_, SO_2_, CO, O_3_ and PM_2.5_) as well as the concurrent day’s average relative humidity [25]. Finally, we assessed confounding for the daily count of influenza ER visits only where the daily number of ER visits for respiratory disease was used as the main outcome variable. Evaluation of confounding for air pollutants, influenza and relative humidity was done by a backward deletion approach [26]. This was done by adjusting for all potential confounders and then by removing one by one in a stepwise manner the least significant confounding variables as long as the total proportional change in effect estimates compared with the fully adjusted model was less than 10%. Covariates that were not confounders, but increased the precision of the estimates were kept in the final model.

Effect estimates were expressed as relative risks (RRs) with 95% confidence intervals (CIs) that can be interpreted as the relative increase in daily ER visits at a specific temperature compared to a temperature reference point for persons with the medical condition under consideration. The Akaike's Information Criterion for quasi-Poisson (Q-AIC) was used to verify the optimal *df* of the natural cubic spline for seasonal effects, non-linear temperature effects and lagged effects [27]. All the analyses were performed using R software (version 3.0.1) with the DLNM package [22].

The effect of extreme hot temperature was evaluated by comparing the relative increase in daily ER visits of the 99^th^ percentile of the daily mean temperature distribution over the study period to the 75^th^ percentile. Extreme cold temperature effect was examined by evaluating the RR of daily ER visits associated with the 1^st^ percentile of temperature relative to the 25^th^ percentile of temperature. These cutpoint comparisons were chosen based on a previous Canadian investigation [25] in order to compare extreme temperature values to normal warm and cold temperatures. Relative increase in daily ER visits were evaluated over cumulative lag periods (i.e. 0 to 1, 0 to 13) in order to capture the possible overall short term and delayed effects of extreme temperatures on adverse health outcomes [28].

Ethics approval for this study has been obtained through a data sharing agreement between the Public Health Agency of Canada and the Canadian Institute for Health Information.

## Results

**Figure 1:**
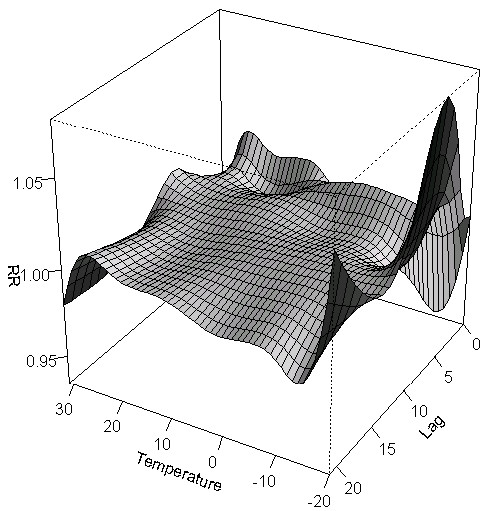
Three-dimensional graph of the relative risks of emergency department visits for cardiovascular diseases by temperature (°C) and lag days in Toronto, Canada.

**Figure 2:**
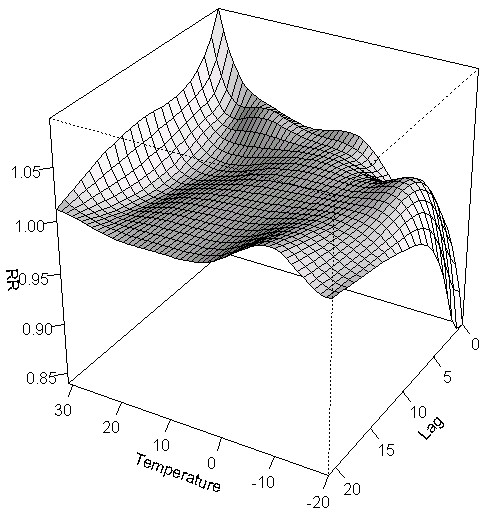
Three-dimensional graph of the relative risks of emergency department visits for respiratory diseases by temperature (°C) and lag days in Toronto, Canada.

Table 1 shows that the study period average of the daily mean temperature was 8.7°C (range: -20.3°C to 31.5°C) and also shows a summary of each pollutant’s levels. Pearson correlation coefficients showed that CO (r = -0.11), SO_2_ (r = -0.07) and NO_2_ (r = -0.20) were negatively correlated with mean temperature (p values < 0.001) while O_3_ (r = 0.34) and PM_2.5_ (r = 0.42) were positively correlated with mean temperature (p values < 0.001). Pearson correlation coefficients ranged from -0.07 to 0.58 when investigating collinearity between air pollutants (p values < 0.001). In addition, all models described below were adjusted for seasonal effects and long term trends, day-of-the-week, relative humidity, daily number of ER visits for influenza (only in the respiratory disease models), nitrogen dioxide (NO_2_), carbon monoxide (CO) and ozone (O_3_) based on the best model fit. A total of 292,666 and 562,738 ER visits for cardiovascular and respiratory diseases, respectively, occurred during the study period in Toronto (Table 2). The most frequent comorbid health conditions for cardiovascular disease ER visits were diabetes (7.3%) and cardiac diseases (6.5%) while respiratory infections (4.0%) represented the most frequent comorbid health condition among respiratory ER visits. In appendix, Figures 1 and 2 are showing three-dimensional graphs of the relative risks of emergency department visits for cardiovascular and respiratory diseases by temperature (°C) and lag days. These graphs show that the effects of hot temperatures on emergency room admissions are generally seen within a few days of the hot day while the effects of cold temperatures are more delayed in time.

**Table 1:** **Summary statistics of weather conditions and air pollution data in Toronto from April 1**^**st **^**2002 to March 31**^**st **^**2010**

**Variables**	**Number of days of measurements**	**Mean**	**Standard deviation**	**Minimum**	**Percentiles**			**Maximum**
					**25**^**th**^	**50**^**th**^	**75**^**th**^	
Mean temperature (°C)	2922	8.7	10.7	-20.3	0.3	9.0	18.2	31.5
Mean relative humidity (%)	2922	69.3	14.4	-25.6	61.9	70.5	78.3	98.8
PM_2.5_ (Ug/m^3^)	2922	8.4	6.85	0	3.8	6.3	10.5	49.2
NO_2_ (Ug/m^3^)	2922	20.5	7.7	5.0	15.0	19.4	24.8	62.6
O_3_ (ppb)	2920	22.1	12.3	2.2	14.6	21.0	28.0	248.0
CO (Ug/m^3^)	2915	0.4	0.2	0	0.2	0.3	0.5	1.8
SO_2_ (Ug/m^3^)	2922	2.2	2.1	0	1.0	1.5	3.0	17.3
Cardiovascular admissions	2922	100.2	20.9	49.0	84.0	101.0	116.0	161.0
Respiratory admissions	2922	192.6	62.8	93.0	156.0	182.0	213.0	801.0

**Table 2:** Number and percentage of cardiovascular and respiratory disease emergency department visits by pre-existing medical conditions

**Comorbid medical condition**	**Cardiovascular disease emergency room visits (n = 292,666)**		**Respiratory disease emergency room visits (n = 562,538)**	
	**No. of admissions**	**%**	**No. of admissions**	**%**
Diabetes	21,386	7.3	11,690	2.1
Respiratory diseases	2,486	0.8	12,915	2.3
Respiratory infections	354	0.1	22,764	4.0
Hypertension	10,932	3.7	2,935	0.5
Cancer	1,926	0.7	2,862	0.5
Kidney diseases	3,109	1.1	1,357	0.2
Pneumonia	2,525	0.9	8,576	1.5
Cardiac diseases	19,167	6.5	6,789	1.2

The cumulative short term effect (i.e. concurrent day and previous day exposure) of extreme hot temperatures on daily ER visits for cardiovascular and respiratory diseases is presented in Table 3. Results show an increased risk of cardiovascular ER visits for persons with comorbid diabetes (RR = 1.13; 95% CI: 1.01 – 1.26) with statistically significant effect modification (REM = 1.12; 95% CI: 1.01 – 1.27). As well, stronger associations were found for subjects with comorbid respiratory disease (REM = 1.17; 95% CI: 1.02 – 1.44)) and cancer (REM = 1.20; 95% CI: 1.02 – 1.49) on respiratory disease ER visits compared with individuals without these conditions. Table 3 also shows the effect of extreme hot temperatures on daily ER visits for cardiovascular and respiratory diseases cumulated over a two-week period. None of the results presented were statistically significant, but some tendency towards increased risk for certain comorbid conditions requires further investigations.

**Table 3:** **Relative risks (RRs)**^**1, 2 **^**and 95% confidence intervals (CIs) for the effect of extreme hot**^**3 **^**temperatures on daily emergency room visits for cardiovascular and respiratory diseases by comorbid health conditions cumulated over specific lag periods in Toronto, Canada**

**Lag period**	**Comorbid medical condition**	**Cardiovascular disease emergency room visits**		**REM**^**4 **^**index (95% CI)**	**Respiratory disease emergency room visits**		**REM**^**4 **^**index (95% CI)**
		**RR**	**95% CI**		**RR**	**95% CI**	
Lag 0 – 1 day	Diabetes	**1.13**	**1.01 – 1.26**	**1.12 (1.01 – 1.27)**	1.04	0.89 – 1.22	1.00 (0.85 – 1.18)
	Respiratory diseases	0.92	0.68 – 1.23	0.90 (0.67 – 1.22)	**1.22**	**1.01 – 1.46**	**1.17 (1.02 – 1.44)**
	Respiratory infections	0.56	0.17 – 1.88	0.55 (0.19 – 2.10)	0.95	0.82 – 1.11	0.91 (0.78 – 1.07)
	Hypertension	1.05	0.88 – 1.25	1.03 (0.86 – 1.23)	1.12	0.82 – 1.53	1.08 (0.79 – 1.48)
	Cancer	0.89	0.60 – 1.32	0.87 (0.59 – 1.30)	**1.25**	**1.04 – 1.50**	**1.20 (1.02 – 1.49)**
	Kidney diseases	1.14	0.82 – 1.58	1.12 (0.81 – 1.57)	1.15	0.77 – 1.71	1.11 (0.75 – 1.67)
	Pneumonia	0.91	0.62 – 1.33	0.89 (0.61 – 1.31)	1.09	0.90 – 1.32	1.05 (0.86 – 1.28)
	Cardiac diseases	0.98	0.86 – 1.12	0.96 (0.84 – 1.10)	1.10	0.91 – 1.33	1.06 (0.87 – 1.29)
Lag 0 – 13 days	Diabetes	1.08	0.74 – 1.64	1.05 (0.69 – 1.59)	0.85	0.47 – 1.52	0.75 (0.42 – 1.38)
	Respiratory diseases	1.09	0.38 – 3.10	1.06 (0.37 – 3.05)	1.42	0.83 – 2.42	1.27 (0.78 – 2.33)
	Respiratory infections	N.E.	-	-	0.95	0.64 – 1.43	0.84 (0.55 – 1.27)
	Hypertension	1.26	0.75 – 2.11	1.22 (0.74 – 2.14)	1.03	0.32 – 3.33	0.92 (0.28 – 2.97)
	Cancer	1.44	0.46 – 4.54	1.40 (0.48 – 4.76)	1.00	0.35 – 2.89	0.88 (0.30 – 2.54)
	Kidney diseases	0.85	0.32 – 2.25	0.82 (0.31 – 2.21)	0.66	0.15 – 2.94	0.58 (0.14 – 2.78)
	Pneumonia	1.82	0.63 – 5.32	1.77 (0.75 – 6.44)	1.00	0.49 – 2.07	0.89 (0.42 – 1.82)
	Cardiac diseases	0.92	0.63 – 1.33	0.89 (0.61 – 1.33)	0.92	0.47 – 1.80	0.81 (0.41 – 1.60)

1. RRs were adjusted for seasonal effects and long term trends, day-of-the-week, relative humidity, daily number of ER visits for influenza (only in the respiratory disease models), nitrogen dioxide (NO2), carbon monoxide (CO) and ozone (O3).

2. RRs represent the relative increase in emergency room visits for cardiovascular and respiratory diseases for persons with a pre-existing medical condition.

3. Hot temperature effects represent the comparison in daily counts of emergency room visits for the 99^th^ percentile of temperature (28.4°C) compared to the 75^th^ percentile of temperature (18.2°C).

4. Relative effect modification.

†Statistically significant results are in bold font.

N.E. (non-estimable).

Similarly, results presented in Table 4 did not reach statistical significance which may be related with study power issues. However, some results will require further clarification. In particular, persons with comorbid kidney diseases may be vulnerable to the cumulative short term effect of extreme cold temperatures on cardiovascular disease ER visits (RR = 1.34; 95% CI: 0.92 – 1.95). Results for the cumulative effects over a two-week period of extreme cold temperatures on cardiovascular and respiratory disease ER visits are also presented in Table 4. Stronger associations for persons with comorbid cardiac diseases (REM = 1.47; 95% CI: 1.06 – 2.23) and kidney diseases (REM = 2.43; 95% CI: 1.59 – 8.83) were observed. Other estimates were not statistically significant.

**Table 4:** **Relative risks (RRs)**^**1, 2 **^**and 95% confidence intervals (CIs) for the effect of extreme cold**^**3 **^**temperatures on daily emergency room visits for cardiovascular and respiratory diseases by comorbid health conditions cumulated over specific lag periods in Toronto, Canada**

**Lag period**	**Comorbid medical condition**	**Cardiovascular disease emergency room visits**		**REM**^**4 **^**index (95 % CI)**	**Respiratory disease emergency room visits**		**REM**^**4 **^**index (95 % CI)**
		**RR**	**95 % CI**		**RR**	**95 % CI**	
0 – 1 day	Diabetes	0.97	0.86 – 1.10	1.01 (0.89 – 1.15)	1.06	0.91 – 1.25	1.15 (0.98 – 1.35)
	Respiratory diseases	0.73	0.50 – 1.07	0.76 (0.54 – 1.16)	1.11	0.94 – 1.33	**1.21 (1.01 – 1.44)**
	Respiratory infections	0.66	0.25 – 1.78	0.68 (0.28 – 1.98)	0.86	0.74 – 1.00	0.92 (0.80 – 1.09)
	Hypertension	0.87	0.72 – 1.04	0.90 (0.34 – 2.42)	1.08	0.72 – 1.61	1.16 (0.78 – 1.74)
	Cancer	0.74	0.48 – 1.15	0.77 (0.52 – 1.24)	1.00	0.65 – 1.50	1.09 (0.71 – 1.65)
	Kidney diseases	1.34	0.92 – 1.95	1.40 (1.00 – 2.13)	1.00	0.62 – 1.61	1.09 (0.67 – 1.75)
	Pneumonia	0.77	0.53 – 1.12	0.80 (0.57 – 1.20)	1.00	0.82 – 1.22	1.09 (0.88 – 1.33)
	Cardiac diseases	1.09	0.94 – 1.26	1.14 (0.98 – 1.33)	1.00	0.81 – 1.23	1.09 (0.89 – 1.34)
0 – 13 days	Diabetes	1.03	0.70 – 1.50	1.13 (0.75 – 1.69)	1.41	0.86 – 2.29	1.47 (0.95 – 2.60)
	Respiratory diseases	0.65	0.22 – 1.90	0.71 (0.26 – 2.26)	1.28	0.83 – 1.99	1.33 (0.88 – 2.17)
	Respiratory infections	0.31	0.02 – 4.94	0.34 (0.03 – 8.57)	1.25	0.80 – 1.95	1.30 (0.84 – 2.12)
	Hypertension	1.03	0.70 – 1.50	1.11 (0.73 – 1.660	0.90	0.31 – 2.64	0.55 (0.32 – 2.77)
	Cancer	0.38	0.09 – 1.52	0.41 (0.14 – 2.39)	1.19	0.41 – 3.42	1.24 (0.43 – 3.66)
	Kidney diseases	**2.24**	**1.12 – 6.10**	**2.43 (1.59 – 8.83)**	1.09	0.27 – 4.29	1.14 (0.28 – 4.56)
	Pneumonia	1.36	0.48 – 3.82	1.48 (0.55 – 4.42)	0.78	0.44 – 1.40	0.81 (0.46 – 1.51)
	Cardiac diseases	**1.35**	**1.02 – 2.04**	**1.47 (1.06 – 2.23)**	1.04	0.55 – 1.97	1.07 (0.56 – 2.05)

1. RRs were adjusted for seasonal effects and long term trends, day-of-the-week, relative humidity, daily number of ER visits for influenza (only in the respiratory disease models), nitrogen dioxide (NO2), carbon monoxide (CO) and ozone (O3).

2. RRs represent the relative increase in emergency room visits for cardiovascular and respiratory diseases for persons with a pre-existing medical condition.

3. Cold temperature effects represent the comparison in daily counts of emergency room visits for the 1^st^ percentile of temperature (-14.4°C) compared to the 25^th^ percentile of temperature (0.3°C).

4. Relative effect modification.

† Statistically significant results are in bold font.

## Discussion

This study showed that persons with comorbid diabetes were vulnerable to the short term effects of extreme hot temperature and consequently had increased risk of being admitted to the emergency department for a cardiovascular event. As well, subjects with comorbid respiratory disease and cancer were found to have increased risk of respiratory disease emergency room visits during short term extreme hot temperature episodes. Finally, the effect of extreme cold temperatures on cardiovascular disease ER visits was positive when cumulated over a two-week period for persons with comorbid cardiac diseases and kidney diseases.

Diabetics were found to be particularly vulnerable to extreme heat with increased risk for adverse cardiovascular events. The underlying mechanism may be related to impaired thermoregulation due to reduced autonomic control and endothelial function during an extreme heat episode [6,12,13]. Previous studies have found increased risk of mortality for persons with diabetes during extreme heat episodes [6,12,13]. Our study also confirmed previous findings that diabetics do not seem to be vulnerable to extreme cold temperatures [6,12,13].

The short term exposure to extreme hot temperatures also increased the risk of respiratory ER visits for subjects with comorbid chronic respiratory diseases. This can be explained by the fact that hot temperatures can affect airways and consequently induce systemic inflammation which can lead to dyspnea and can be a particular issue among people who already have frail respiratory conditions [29]. However, we did not find that persons with a comorbid chronic respiratory disease had increased risk of respiratory disease ER visits during extreme cold temperatures as previously reported [13]. However, we may not have had enough power to detect this association in this study.

Subjects diagnosed with cancer in the year preceding their admission to the emergency department were found to have an increased risk of respiratory ER visits during an episode of extreme heat. This result could be explained by the fact that cancer patients may already suffer from dyspnea as a side effect from chemotherapy and radiotherapy treatments [30]. Thus, the increasing demand of extreme heat on airways combined with shortness of breath among cancer patients could increase their risks of an adverse respiratory event. To our knowledge, this finding has not been previously reported which requires further investigations.

Our study also showed that extreme cold temperatures over a two-week period increased the risk of ER visits for cardiovascular diseases among patients with comorbid cardiac diseases. Biological mechanisms that can lead to a cardiovascular disease event include the fact that cold temperatures are associated with an increase in systolic and diastolic blood pressures, serum low density lipoprotein cholesterol concentration, heart rate, plasma fibrinogen concentrations, platelet viscosity and peripheral vasoconstriction as well as a decrease in high density lipoprotein cholesterol level which may place persons with comorbid cardiac diseases in a vulnerable state [31-33]. In fact, it’s been previously shown that subjects who had a prior acute myocardial infarction were more sensitive to extreme cold temperatures [7].

The biologic mechanism underlying the relation of extreme cold temperature and cardiovascular disease ER visit among patients with comorbid kidney diseases may be related to the fact that renal disorders are known to increase blood pressure [34]. The added effect of extreme cold temperatures which are also associated with an increase in blood pressure [33] may act synergistically among persons with renal disorders in developing an adverse cardiovascular disease. This hypothesis requires further clarification.

There are some methodological limitations in this study. First, we relied on fixed-site monitoring stations for daily weather and air pollution data rather than measuring individual exposures. However, these measurement errors are likely to be random which would usually result in an underestimation of the relative risks [35]. In addition, results of this study may be affected by factors that are specific to the city of Toronto which can modify the effect of temperature on cardiorespiratory morbidity (e.g. social factors, demography, infrastructural factors, housing characteristics and access to air conditioning) [36]. This study may also lack statistical power to evaluate the effects of extreme temperatures in subgroups of comorbid conditions. We also investigated the risk only among subgroups and did not calculate a proper statistical value to evaluate the effect modification of each comorbid condition in the relation between extreme temperatures and cardiorespiratory morbidity. Our case definition of several comorbid health conditions relied only on secondary diagnoses rather than identifying these conditions using a combination of health administrative databases (e.g. outpatient visits, hospitalizations, emergency room visits).

The strengths of our study include the relatively long study period using ER data with a substantial amount of admissions and the use of a flexible statistical approach to examine distributed lag and non-linear effects of temperatures on cardiorespiratory morbidity. In addition, we adjusted for a range of confounders including relative humidity, air pollutants and influenza.

## Conclusion

The results of this study showed that extreme temperatures in Toronto were associated with increased risk of ER visits for cardiorespiratory diseases in subgroups with underlying medical conditions. The results from this study provide evidence to strengthen the need for public health decision makers to consider the impact of extreme temperatures according to comorbidity status.

## Consent

Written informed consent was obtained from the patient for the publication of this report and any accompanying images through a data sharing agreement between the Public Health Agency of Canada and the Canadian Institute for Health Information.

## Abbreviations

ER: Emergency room; RR: Relative risk; CI: Confidence interval; ICD-10: International classification of disease, tenth revision; NACRS: National Ambulatory Care Reporting System; CIHR: Canadian Institute for Health Research; PHAC: Public Health Agency of Canada; NAPS: National Air Pollution Surveillance Network; NO2: Nitrogen dioxide; SO2: Sulphur dioxide; CO: Carbon monoxide; O3: Ozone; PM2.5: Particulate matter of median aerodynamic diameter less than 2.5 microns; Q-AIC: Akaike's Information Criterion for quasi-Poisson; Df: Degree of freedom; DLNM: Distributed lag non-linear model; GLM: Generalized linear model; DLNM: Distributed lag non-linear model (DLNM).

## Competing interests

The authors declare that they have no competing interests.

## Authors’ contributions

EL, AG and SC were responsible for study concept and design and supervised the study. EL and XW acquired the data, which was analyzed and interpreted by EL, AG, XW, HC, AY, MDF and SC. EL drafted the manuscript, which was critically revised for important intellectual content by EL, AG, XW, HC, AY, MDF and SC. XW did the statistical analysis with supervision from AG and EL. EL is the guarantor. All authors read and approved the final manuscript.
